# The combined effect of EGR and hydrogen addition on a *Syzygium cumini* (jamun) liquid biofuel engine

**DOI:** 10.1186/s13068-023-02330-2

**Published:** 2023-06-24

**Authors:** Chandrasekar Kannappan, Sudhakar Sengottaiyan, Rajappan Ramasamy

**Affiliations:** 1 Research Scholar, Department of Mechanical Engineering, Annamalai University, Chidambaram, 608002 Tamil Nadu India; 2 Department of Mechanical Engineering, Annamalai University, Chidambaram, 608002 Tamil Nadu India; 3 Department of Mechanical Engineering, Mailam Engineering College, Tindivanam, India

**Keywords:** Diesel fuel, *Syzygiumcumini* (jamun), Hydrogen fuel, EGR, NO_X_ emissions

## Abstract

Rapid depletion of fossil fuels required the development of alternate and sustainable fuel sources that could replace conventional fuel while having no negative environmental impact. Combining hydrogen induction with biodiesel ensures strict emission standards and lowers energy consumption compared to conventional fuels. In this study, the performance, emissions, and combustion of a CI engine for *Syzygium* *cumini* (B20) were assessed and compared to diesel fuel while using a fixed amount of hydrogen flow rate (6L/m). Throughout the experiment, an exhaust gas recirculation (EGR) technology of 10% and 20% and a constant engine speed of 1500 rpm at varying engine load circumstances were used. When hydrogen is added to B20, it decrease the emissions of carbon monoxide (CO), unburned hydrocarbons (UHC), brake thermal efficiency (BTE), and brake specific energy consumption (BSEC). At maximum load, the use of the EGR system decreased the exhaust gas temperature (EGT) by 13.4% and nitrogen oxide (NO_X_) emission by 25%, but it had a negative impact on BTE, BSEC, as well as other emission parameters including CO and UHC. Therefore, using hydrogen in dual fuel mode in a CI engine enhances performance and lowers exhaust emissions, while using the EGR approach reduces NO_X_ emissions.

## Introduction

The world’s population is still growing and industrialised and developing nations continue to have a high demand for energy. Fossil fuels are currently being utilised at an increasing rate, which has led to the formation of two serious crises: the exhaustibility of fossil fuels and the combustion products that result from those fuels, both of which are contributors to the current state of the environment. Due to their accessibility and practical use, fossil fuels provided over 70% of the world’s energy production demands in 2020. The availability was predicted to reach its peak soon and then start to decline [1]. Even if countries all over the world pay attention to renewable energy sources, the contribution at the moment is not that different. 2019’s global campaign against the Coronavirus Disease is likely to draw a lot of attention to the impact that burning fossil fuels has on the environment (COVID-19). Despite the darkness caused by this pandemic, there has been a surprising environmental benefit: air pollution has dramatically decreased over a significant portion of the earth. China's Ministry of Ecology and Environment reports that air quality has improved 21% over the course of the past year as of February 2020 [2]. This notable drop in emissions has been brought about by decreased vehicle traffic and the closure of industrial operations as a result of people staying at home to prevent the virus from spreading [3].

Although several NOx control techniques are built into the engine, the EGR is still the most efficient technological approach since it recycles exhaust gas into the engine's intake manifold, reducing nitrogen oxide emissions from diesel engines. This is feasible because it lowers the flame temperature and oxygen content in the working fluid in the combustion chamber. Researchers have employed the EGR technology to lower NOx emissions when using biodiesel or diesel engines that have been hydrogen-enriched [4].

A small amount of hydrogen was employed as the inducted fuel by Kumar et al. [5], whereas diesel and jatropha oil were used as pilot fuels in a diesel engine. The authors reported an improvement in hydrogen production through an improvement in brake thermal efficiency, lower smoke level, UHC, and CO emissions, and an increase in NO_X_ emission.

In a diesel engine that ran on standard diesel and 100% waste cooking oil methyl ester, Nanthagopal et al. [6] used 30% EGR. According to the study, the 30% EGR addition reduced BTE and NOx. Additionally, the emissions of carbon monoxide and unburned hydrocarbons for both fuels at load have significantly increased due to the 30% EGR.

In a diesel engine using the exhaust gas recirculation (EGR) technology, Saravanan et al. [7] employed hydrogen-enriched air as the intake charge with a hydrogen flow rate of 20 L per minute (lpm). Lower specific energy consumption, smoke level, particle and NO_X_ emissions were produced by using hydrogen in dual fuel mode with EGR.

In the dual fuel mode engine for hydrogen and diesel, the inventor also introduced the EGR [5]. Here, the EGR percentages were kept at 10% and 20% while the H_2_ fuel flow was adjusted from 2 to 10 lpm at a pressure of 2 bar. Based on the findings, the ideal hydrogen flow rate combination of 6 lpm was shown to decrease BTE, HC, and CO while increasing NOx emission in the absence of EGR. High NOx emission was decreased using 10% and 20% EGR.

Through the diesel engine's input manifold, Probir Kumar Bose and Dines Maji [8] incorporated hydrogen along with EGR and air. With a 2 bar injection pressure, the researchers kept the hydrogen injection rates at five different levels: 2 L/min, 4 L/min, 6 L/min, 8 L/min, and 10 L/min. The findings suggested that when the EGR rate was raised, the HRR and peak pressure decreased.

According to the studies mentioned above, biodiesel is a reliable alternative fuel to fossil fuels. The use of hydrogen enhancement and EGR technologies for diesel–biodiesel blended fuel in engines to reduce NOX and other pollutants is quite uncommon, nevertheless. Furthermore, research on third generation biodiesel is still in the early stages and is only being done at the production level.

These barriers to biodiesel use in cars can be overcome by using biodiesel instead of gaseous fuels like hydrogen and natural gas. The gaseous fuels’ rapid flame and mixture creation improve combustion efficiency while also reducing emission issues. However, the drawback of using gaseous fuels is that they result in higher NOx emissions and lower power production.

Currently, EGR technology reduces NOx emissions from diesel engines. The majority of literature discusses diesel–hydrogen dual fuel engines with EGR. The use of EGR in biodiesel–hydrogen dual fuel engines to reduce NOx emissions has not been the subject of any substantial research, though. Moreover, compared to diesel fuel, the cost of manufacturing biodiesel from various edible and non-edible oils is considerable.

In this study, several EGR percentages were used to reduce NO_X_ emission. The performance, combustion, and emission characteristics of the dual fuel engine were examined using the improved *Syzygium* *cumini* (jamun) biodiesel–hydrogen–EGR system.

## Materials, equipment and methodology

### Storing hydrogen using safety equipment

A flame tap is used in the supply line that prevent backfire and also helps to reduce the explosions inside the setup. When an unfavourable combustion phenomenon takes place, flashback arrestors are employed to restrain or stop the flow of gas and put out the flame before it can reach the gas source [9]. Different non-reverse control valves were installed in the fuel delivery line to prevent gas backflow into the engine cylinder.

### Process of inducing hydrogen

Hydrogen can be delivered from the cylinder to the engine's combustion chamber using either the air intake manifold system or a direct hydrogen injection system [10]. Again, there are two methods for adding hydrogen to the intake manifold system: either by utilising a gas carburetor for carburation or by using a time port injection system (TPI). This technique employed a gas carburetion induction system to provide hydrogen into the intake manifold. The major fuel source was a diesel–biodiesel mix (B20) containing a certain amount of *Syzygium* *cumini* (jamun) biodiesel (20% v/v). Using a fuel injector, the engine received a conventional injection of the diesel–biodiesel mixture.

### Exhaust gas recirculation (EGR)

**Fig. 1:**
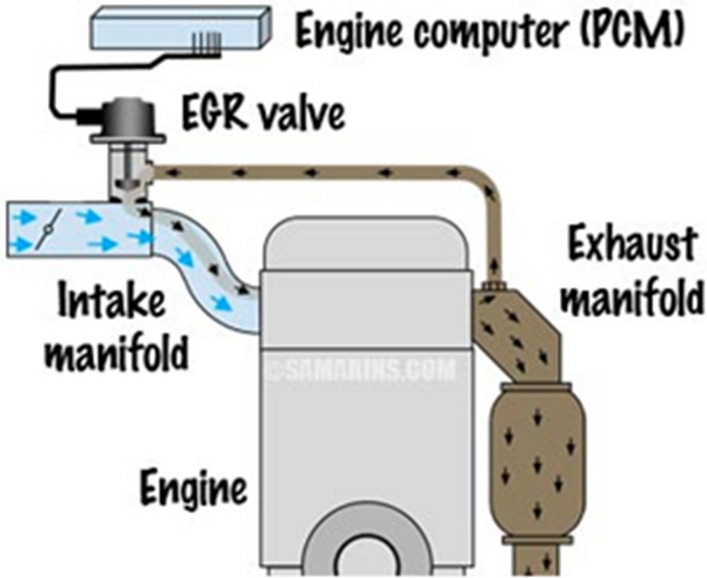
Schematic arrangement of EGR operation

The exhaust gases from the engine cylinders were partially recirculated into the intake manifold by building an exterior EGR pipeline. It was kept at a distance that allowed for a complete mixing of exhaust gases and fresh air. The gases were sent through a particulate filter made of steel wool before entering the cylinder (see Fig. 1) [11] to prevent too many particles from entering the combustion chamber. The EGR percentage is calculated using the formula below:

$${\text{EGR rate}}\left( \% \right) \, = \, \frac{{{\text{Q}}_{{\text{without EGR}}} - {\text{Q}}_{{\text{with EGR}}} }}{{_{{{\text{Q}}_{{\text{without EGR}}} }} }},$$

where Q without EGR stands for the airflow rate prior to EGR and Q with EGR for the airflow rate following EGR. The EGR percentages were changed in increments of 10% and 20%.

### Materials (sample collection)

**Fig. 2:**
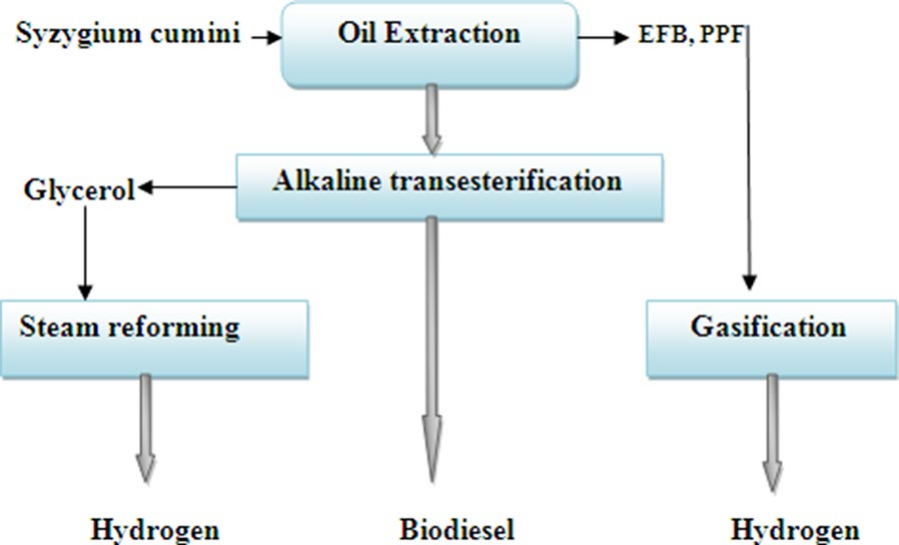
The overall layout of the biodiesel mixed biomass biorefinery

The general configuration of the collective biomass biorefinery using oils from *Syzygium* *cumini* (jamun) is shown in Fig. 2. A large amount of lignocellulose wastes, which can be used to create high-value goods, can be produced by the extraction of both oils. The proposed biorefinery would use these lignocellulose materials to gasify them and create hydrogen. The glycerol generated during the transesterification process is also taken into account for steam reforming energy generation. In this sense, the collaborative biorefinery's conceptual design is divided into four main sections: I the production of biodiesel from oils through alkaline transesterification; II the reforming of glycerol using steam; III the gasification of biomass; and IV the purifying of hydrogen [12] (see Table 1).

**Table 1:** Hydrogen, *Syzygium* *cumini* (jamun), and diesel fuel properties

S. no.	Property	Diesel	Hydrogen	*Syzygium* *cumini* (jamun)
1	Molecular weight (gm/mol)	160	1.987	918.54
2	Stoichiometric air fuel ratio	33.5	13.8	N/A
3	Flame velocity (cm/s)	27	260	34
4	Auto-ignition temperature (K)	534	567	700–830
5	Heat of combustion (kJ/kg)	40.3	125	64
6	Density of gas at NTP (g/cm^3^)	0.85	0.078	0.74
7	Octane number	–	120	55
8	Cetane number	45–55	–	45
9	Boiling point (K)	550–630	21.75	340—405
10	Specific gravity	0.78	0.089	0.918

### Experimental set-up

**Fig. 3:**
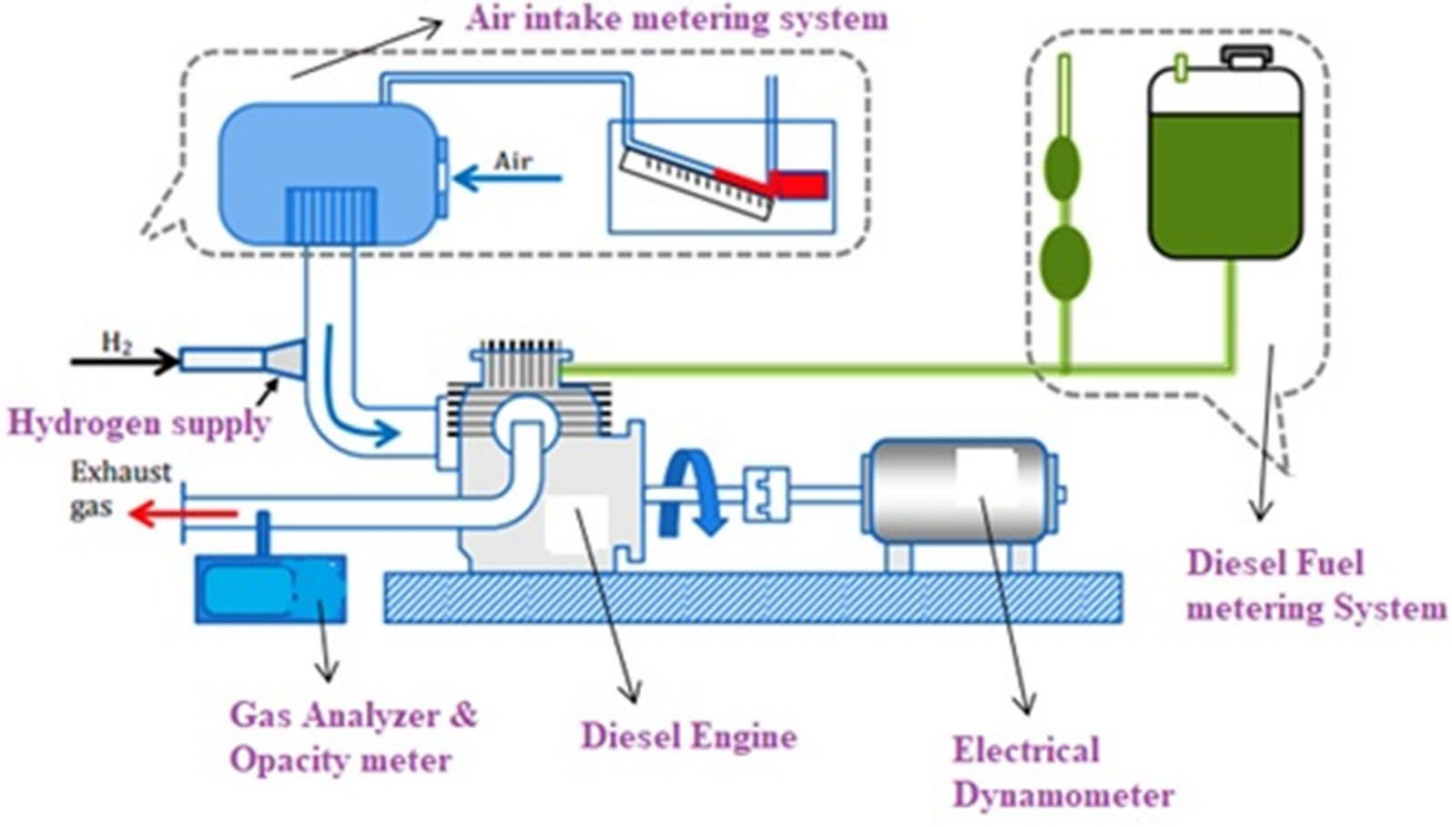
Experimental set-up

The results of experimental research on the co-combustion of hydrogen and biodiesel blends in an internal combustion (IC) engine are summarised in the study. The tests were conducted using an industrial engine with a single cylinder, water cooling, and two valves. A constant 1500 revolutions per minute were made by the engine. Table 2 describes the engine specifications in more detail. Additional fuel supply systems are present in the research engine. Biodiesel was injected directly into the engine's combustion chamber. The duration of the injection stayed unchanged. A dynamometer is part of the measurement system on the test stand. A schematic of a test stand with an engine is shown in Fig. 3. In order to induce a specified volume of exhaust gas into the intake manifold for EGR operation, a fine control valve is often fitted in the EGR loop. The oscillation of a wave of recycled exhaust gas is lessened by the addition of an air box. The flow rate of the recycled exhaust gas was determined using an orifice metre. The desired temperature was then achieved by utilising an EGR cooler to cool the recirculated exhaust gas. A schematic representation of the EGR system is shown in Fig. 3.

**Table 2:** Specification of the engine

Engine make	Kirloskar AV-1
Type	Single cylinder and water cooled
Bore × stroke	80 × 110 mm
Displacement	550 CC
Max. power	3.7 kW at 1500 rpm
Fuel injection timing	23° bTDC
Compression ratio	16.5:1
Loading device	Electrical dynamometer

### Estimation of error and uncertainty

Measurement errors must be present during the experiment and rely on the precision of the equipment, the surroundings, the observations, etc. [13]. This phrase is frequently used to refer to uncertainty, which is a crucial sign of the experiment's precision and validity. Uncertainty can result from random or fixed errors; fixed errors occur during direct measurement and are easy to quantify, but random errors can be calculated as percentage uncertainties of that parameter. Table 3 displays the proportion of uncertainty for a few parameters. The linearised approximation method was used to calculate the individual parameter uncertainty. Then, the overall uncertainty was determined using Eq. (1).

**Table 3:** Uncertainty regarding numerous parameters

Parameters	Uncertainty (%
Load	0.3
Speed	0.2
Pressure	0.4
Brake thermal efficiency	0.6
Crank angle	0.2
Mass flow rate for hydrogen	0.4
Brake specific fuel consumption	0.7
Temperature	0.2
Unburnt hydrocarbon	0.13
Oxides of nitrogen	0.9
Carbon monoxide	0.04

The value $\pm 2\sigma$ is termed as a mean limit in this about 96% of values was measured:

1 $$\Delta {\text{Z}}_{{\text{i}}} \, = \,\frac{{2\sigma_{{\text{i}}} }}{{\overline{{{\text{Z}}_{{\text{i}}} }} }}\, \times \,100.$$

In the above equation, the parameter $\overline{{{\text{Z}}_{{\text{i}}} }}$—the experimental interpretations, ${\text{Z}}_{{\text{i}}}$—a number of readings, ${\upsigma }_{{\text{i}}}$—the value of standard deviation. The estimated uncertainty of various constraints was explored as follows:

2 $$P\, = \,f(Z_{1} ,Z_{2} ,Z_{3} ,Z_{4} ,Z_{5} ,........................Z_{n} ),$$

3 $$\Delta {\text{P}} = \,\sqrt {\,\,\left( {\left[ {\frac{{\partial {\text{P}}}}{{\partial {\text{Z}}_{1} }}\Delta {\text{Z}}_{1} } \right]^{2} + \,\left[ {\frac{{\partial {\text{P}}}}{{\partial {\text{Z}}_{2} }}\Delta {\text{Z}}_{2} } \right]^{2} + \left[ {\frac{{\partial {\text{P}}}}{{\partial {\text{Z}}_{3} }}\Delta {\text{Z}}_{3} } \right]^{2} + \cdots \left[ {\frac{{\partial {\text{P}}}}{{\partial {\text{Z}}_{{\text{n}}} }}\Delta {\text{Z}}_{{\text{n}}} } \right]^{2} } \right)} .$$

In the above equation, the functions ${\text{Z}}_{{1}}$, ${\text{Z}}_{{2}}$, ${\text{Z}}_{{3}}$,………………….${\text{Z}}_{{\text{n}}}$ delivers the number of readings taken from the experiment. Thence, “${\Delta P}$”was calculated by the root mean square and the errors related to the measured limits.

## Results and discussion

In the current exploration, B20 served as the main fuel while hydrogen was used for dual fuel mode operation. The performance and emissions characteristics of B20 with hydrogen enrichment and 10% and 20% EGR technology are also evaluated and contrasted to conventional fuel (see Table 4).

**Table 4:** Effect of biodiesel–diesel blends on engine performance and emissions

Type of engine and test conditions	Feed stock of biodiesel	Performance (compared to diesel)		Emission (compared to diesel)				References
		BTE	BSFC	HC	CO	NOx	Smoke	
Single cylinder, 4 stroke diesel engine at 1500 rpm	Pongamia biodiesel	Decrease	Increases	Increase	Increase	Increase	Increase	Muralidharan et al. [14]
Single cylinder, 4 stroke diesel engine at 1500 rpm	Pongamia biodiesel	Decrease	-	Decrease	Decrease	Increase	Decrease	Rao and Anand [15]
Single cylinder, 4 stroke diesel engine at 1500 rpm	Mahua methyl ester (MOME),	Decrease	-	Decrease	Decrease	Increase	Increase	Nayak et al. [16]
Single cylinder, 4 stroke diesel engine at 2000 rpm	Pongamia methyl ester	-	Decrease	Decrease	Decrease	Increase	-	Thiruvengadaravi et al. [17]
Single cylinder, 4 stroke diesel engine at 1500 rpm	Pongamia methyl ester	Decrease	Increase	Decrease	Decrease	Decrease	-	Perumal and Ilangkumaran [18]
Single cylinder, 4 stroke diesel engine at 1500 rpm	Methyl ester mango seed oil (MEMSO)	Decrease	Increase	Decrease	Decrease	Increase	Decrease	Vijayaraj et al. [19]
Single cylinder, 4 stroke diesel engine at 1500 rpm	Mahua oil ethyl ester (MOEE)	Decrease	Increase	Decrease	Decrease	Decrease	Decrease	Puhan et al. [20]
Multi cylinder turbo charged, diesel engine	Mahua methyl ester	Decrease	Increase	Decrease	Decrease	Increase	Decrease	Godiganur et al. [21]
Single cylinder, 4 stroke diesel engine at 1500 rpm	Mahua biodiesel	Decrease	Increase	–	–	–	–	Raheman et al. [22]
Single cylinder, 4 stroke diesel engine at 1500 rpm	Mahua alkyl ester such as methyl ester, ethyl ester and butyl esters	Decrease	Increase	Decrease	Decrease	Increase	Decrease	Puhan et al. [23]
Single cylinder, 4 stroke diesel engine at 1500 rpm	*Pongamia pinnata* methyl ester (PPME)	Decrease	Increase	Decrease	Decrease	Increase	Decrease	Suresh Kumar et al. [24]
Single cylinder, 4 stroke diesel engine at 1500 rpm	Pongamia, rice bran, sunflower and palm oil	Increase	Decrease	Decrease	Decrease	Increase	Decrease	Tamil selvan et al. [25]
Single cylinder, 4 stroke diesel engine at 1500 rpm	Annona methyl ester (AME)	–	–	Decrease	Decrease	Increase	Decrease	NaruunNabi et al. [26]
Single cylinder, 4 stroke diesel engine at 1500 rpm	Neem oil methyl ester (NOME)	Decrease	Increase	Decrease	Decrease	Increase	Decrease	Senthil et al. [27]
Single cylinder, 4 stroke diesel engine at 1500 rpm	Karaja biodiesel	Decrease (3–5%)	Increase	Decrease	Decrease	Decrease	Decrease	NaruunNabi et al. [28]
Single cylinder, 4 stroke diesel engine at 1500 rpm	Castor biodiesel	Decrease	Increase	Decrease	Decrease	Increase	Decrease	Ismail et al. [29]
Single cylinder, 4 stroke diesel engine at 1500 rpm	Soyabean methyl ester	Decrease	Increase	Decrease	Decrease	Increase	Decrease	Özener et al. [30]
Single cylinder, 4 stroke diesel engine at 1500 rpm	Jatropha methyl ester	Decrease	Increase	Decrease	Decrease	Increase	Decrease	Paul et al. [31]
Single cylinder, 4 stroke diesel engine at 1500 rpm	*Citrus sinensis *biodiesel (CSB)	Decrease	Increase	Decrease	Decrease	Increase	Decrease	Uludamar et al. [32]
Single cylinder, 4 stroke diesel engine at 1500 rpm	Biodiesel	Decrease	Increase	Decrease	Decrease	Increase	Decrease	Lahane et al. [33]
Single cylinder, 4 stroke diesel engine at 1500 rpm	Biodiesel	Decrease	Increase	Decrease	Decrease	Increase	Decrease	Hasan Ali et al. [34]
Single cylinder, 4 stroke diesel engine at 1500 rpm	Corn oil, rapeseed oil and waste oil	Decrease	Increase	Decrease	Decrease	Increase	Decrease	Tesfa et al. [35]

### Performance parameter

#### Brake thermal efficiency (BTE)

**Fig. 4:**
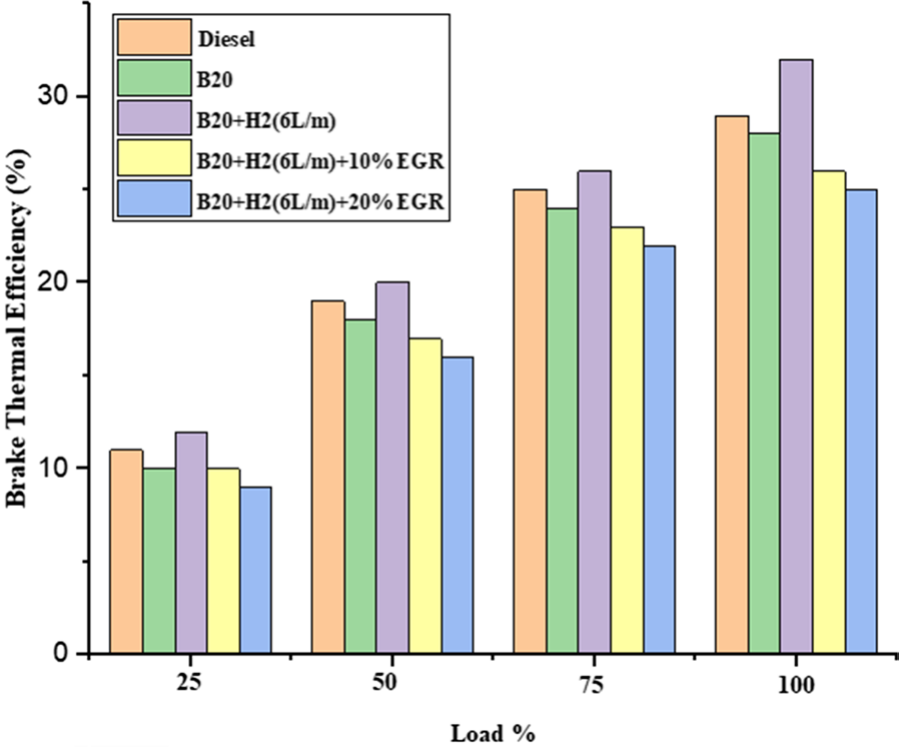
Effect of BTE with load variation

Figure 4 shows the effect of load on BTE. The graph clearly demonstrates that BTE rose by 31.4% in comparison to diesel fuel, which rose by 25.3%, while BTE of B20 and B20 + H2 (6L/m) + 10% EGR was 25% and 24.12%, respectively, at 75% load. BTE was 24.7% for B20, 23.8% for B20 + H2 (6L/m) + 20% EGR, and 24.2% for DF at maximum load. For B20 + H2(6L/m), BTE was 31.4%. The operation's hydrogen enrichment, which enhances fuel combustion, resulted in a rise in BTE [36]. Reduced load range biodiesel presence was accompanied by low hydrogen combustion efficiency. Hydrogen burned with great combustion efficiency when biodiesel was present and the range load was high. EGR was added to all engine loads, which decreased the BTE. This could be explained by the presence of EGR, which lowers the oxygen concentration in intake air and significantly harms combustion [37].

#### Brake specific energy consumption (BSEC)

**Fig. 5:**
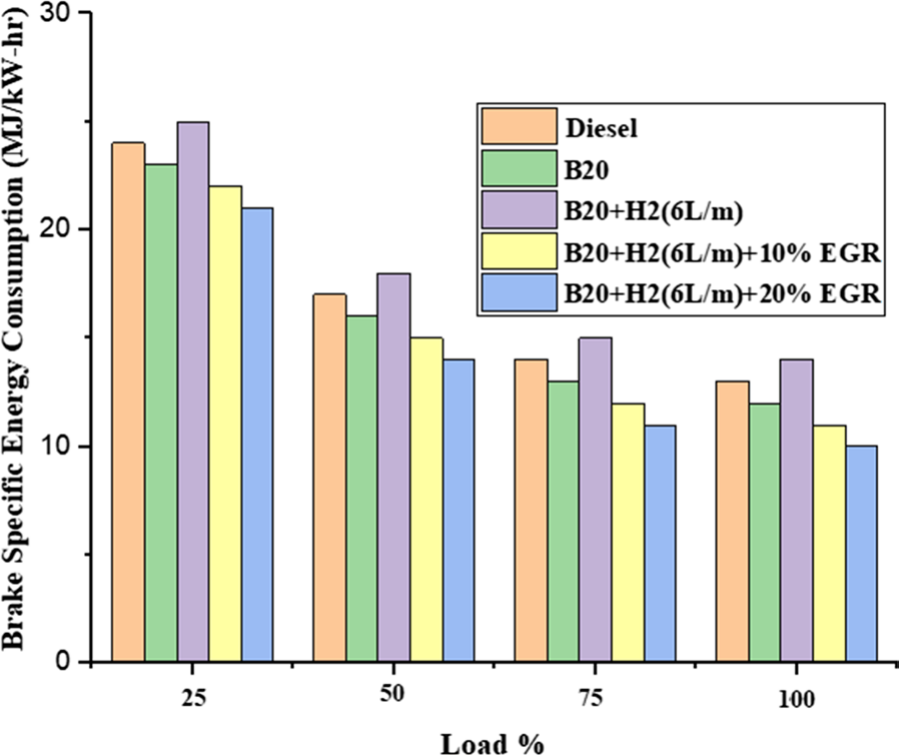
Effect of BSEC with load variation

Figure 5 shows how BSEC varies as a function of load variation and shows how BSEC declines as load increases. While tidy diesel fuel had a BSEC of 26.02 MJ/kW-h, which was 11.6% higher than baseline diesel fuel at 25% load, B20 had a BSEC of 27.84 MJ/kW-h. This was caused by B20’s higher viscosity and lower LCV when compared to diesel fuel. As a result, at the same load situation, the BSEC of B20 + H2 (6L/m) (18.79 MJ/ kW-h) was determined to be 21.6% lower than that of diesel fuel. Under full load conditions, the BSEC of diesel fuel, B20, B20 + H2 (6L/m), B20 + H2 (6L/m) + 10% EGR, and B20 + H2 (6L/m) + 20% EGR were measured as 14.34, 15.05, 16.4, 12.3 and 11.4 MJ/kW-h, respectively. Due to greater air and hydrogen mixing, B20 + H2 burns more efficiently (6L/m), which results in reduced BSEC [38]. Again increasing and having a negative impact on engine combustion when EGR was operating, BSEC (21.04 MJ/kW-h) was measured. By reducing engine speed as a result of incomplete combustion as opposed to when there is no EGR, it results in greater energy consumption.

#### Exhaust gas temperature (EGT)

**Fig. 6:**
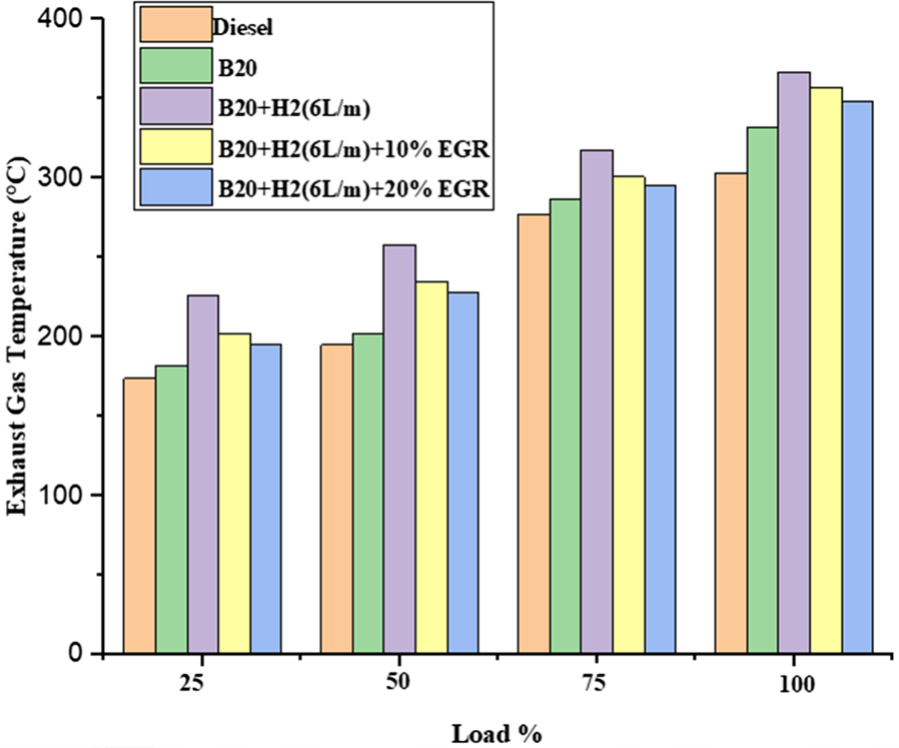
Effect of EGT with load variation

Figure 6 depicts the impact of engine load on EGT for several diesel fuels, including B20, B20 + H2 (6L/m), B20 + H2 (6L/m) + 10% EGR, and B20 + H (6L/m) + 20% EGR.. As loads grew, EGT climbed as well, reaching its maximum value under conditions of full load. At a 100% load condition, the EGT of diesel fuel, B20, B20 + H2 (6L/m), B20 + H2 (6L/m) + 10% EGR, and B20 + H2 (6L/m) + 20% EGR were found to be 185 °C, 195 °C, 240 °C, 230 °C, and 220 °C, respectively. The faster and better fuel combustion that led to the temperature reaching its peak can be blamed for the enhanced EGT [39]. The reduced EGT after EGR operation compared to B20 + H2 (6L/m) was attributed to inefficient fuel combustion caused by the greater specific heat and a lack of enough oxygen in the intake charge.

### Emission parameters

#### Carbon monoxide (CO)

**Fig. 7:**
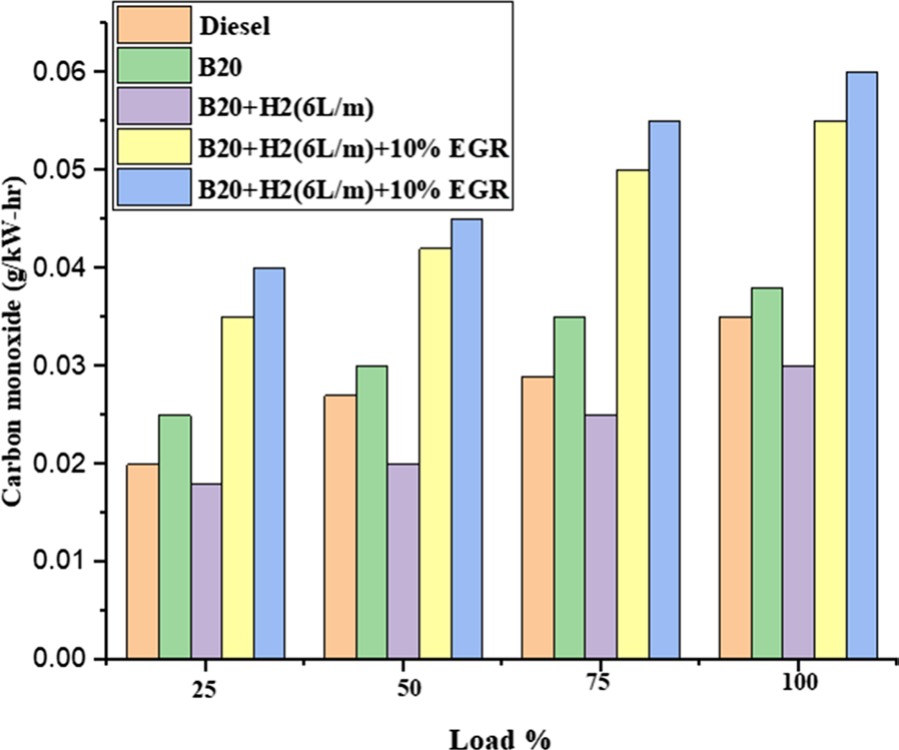
Effect of CO with load variation

Figure 7 depicts the variation in CO emission with load and steady engine speed. Engine load increased while CO decreased. The lack of oxygen and low temperature during combustion are the main causes of CO release. Air intake is considerably lower at lower load conditions compared to higher load conditions, which led to higher CO emission. The introduction of the high EGR percentage resulted in an increase in CO emission [9]. The reaction speed, O2 concentration, and in-cylinder temperature were all found to be reduced as the EGR rate was raised. As a result, more CO was released since the oxidation reaction was weaker. When the EGR was 40% compared to B20 + H2 (6L/m) fuel mode at full load, the percentage of CO increased by up to 30%, as can be shown.

#### HC emission

**Fig. 8:**
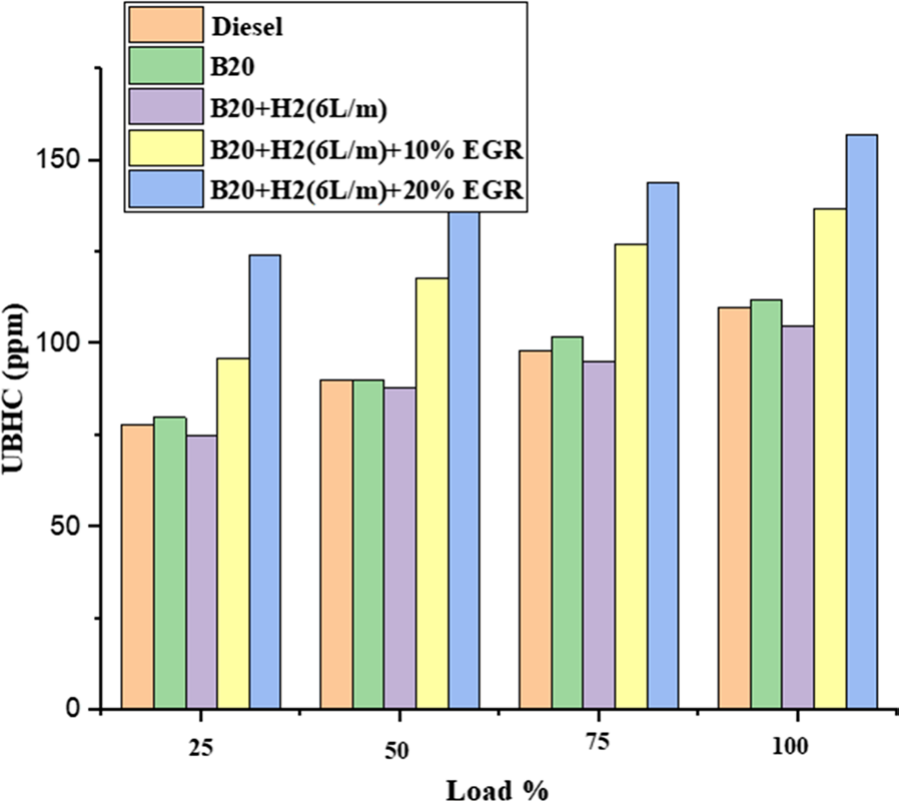
Effect of UHC with load variation

Unburned hydrocarbon (UHC) emission from all evaluated fuels is shown in Fig. 8 as a result of load fluctuation. The image made it very evident that load increased resulted in a decrease in UHC emission. When the engine is not running, the UHC emissions of diesel fuel, B20, B20 + H2 (6L/m), B20 + H2 (6L/m) + 10% EGR, and B20 + H2 (6L/m) + 20% EGR are measured as 65 ppm, 72 ppm, 53 ppm, 84 ppm, and 124 ppm, respectively, whereas when the engine is running at full capacity, they are measured as 113 ppm. There is also the additional finding that using B20 + H2 (6L/m) fuel, the HC emission rose as the EGR percentage increased. Due to the low amount of overly accessible oxygen, rich air fuel mixtures burn more slowly and produce more UHC, which causes a rise in HC. When the EGR was 10% or 20%, the UHC climbed to 15% [40].

#### Nitrogen oxide (NOX)

**Fig. 9:**
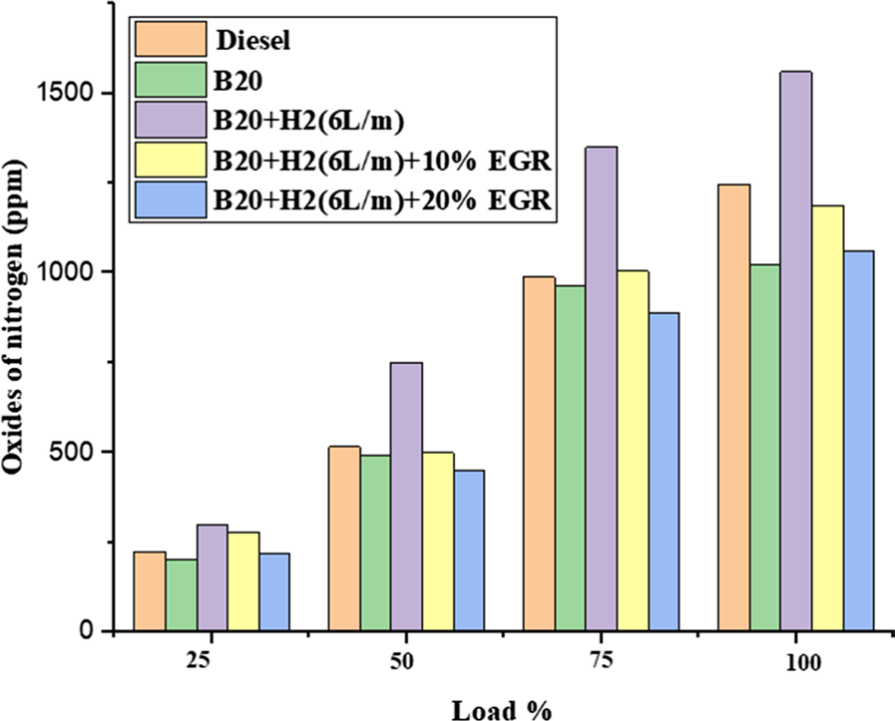
Effect of NOx with load variation

The effect of NOX emission with variable engine load and constant engine speed is depicted in Fig. 9. NOX emission was seen to be at its greatest during hydrogen enhancement for B20 without an EGR system (i.e. B20 + H2 (6L/m). The increased NOX emission at full load varied from 950 to 1450 ppm. This result can be attributed to the increased combustion temperature that enhanced combustion introduced into the combustion chamber [41]. The NOX emission for B20 + H2 (6L/m) + 10% EGR was found to be 1003 ppm, which is lower than the 1450 ppm at 100% load for B20 + H2 (6L/m) without EGR. By reducing the flame temperature during combustion, EGR lowered NOX generation by 20%. This result is due to the intake charge's oxygen concentration being reduced by the recirculating gas. With B20 + H2 (6L/m) gasoline, EGR rates ranging from 10 to 20% were used. EGR rate increases reduce NOx emissions. While the addition of EGR lowers the combustion temperature and subsequently the combustion efficiency, resulting in decreased NOx, the reaction becomes the opposite when the hydrogen is supplemented with the fuel.

### Combustion analysis

#### Cylinder pressure

**Fig. 10:**
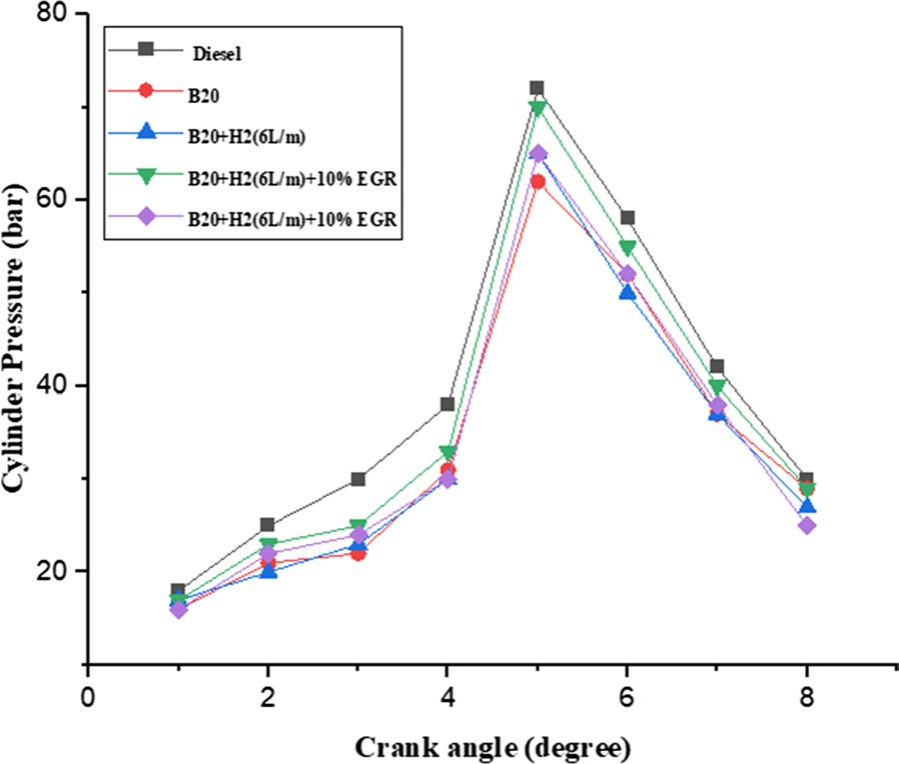
Effect of cylinder pressure with crank angle

In all of the fuels that were evaluated, the cylinder pressure in relation to crank angle diagrams is shown in Fig. 10 under conditions of maximum load. With regard to diesel engines, the amount of fuel burned during the premixed combustion phase has a significant impact on the peak pressure at the start of the combustion rate. At the time of the delay period, the premixed or uncontrolled combustion phase is handled by both the ignition delay period and the preparation of the mixture. For B20, B20 + H2 (6L/m), 10% EGR, and 20% EGR at full load, the peak pressure readings were 74.32, 68.54, 67.35, and 67.14 bars, respectively. As can be seen, compared to other modes, H_2_ enrichment allows for a larger peak pressure. This is as a result of H_2_’s improved combustion and shorter ignition delay time. A key influence is played by the installation of EGR and its emphasis on the peak pressure and ignition delay time [42].

#### Heat release rate

**Fig. 11:**
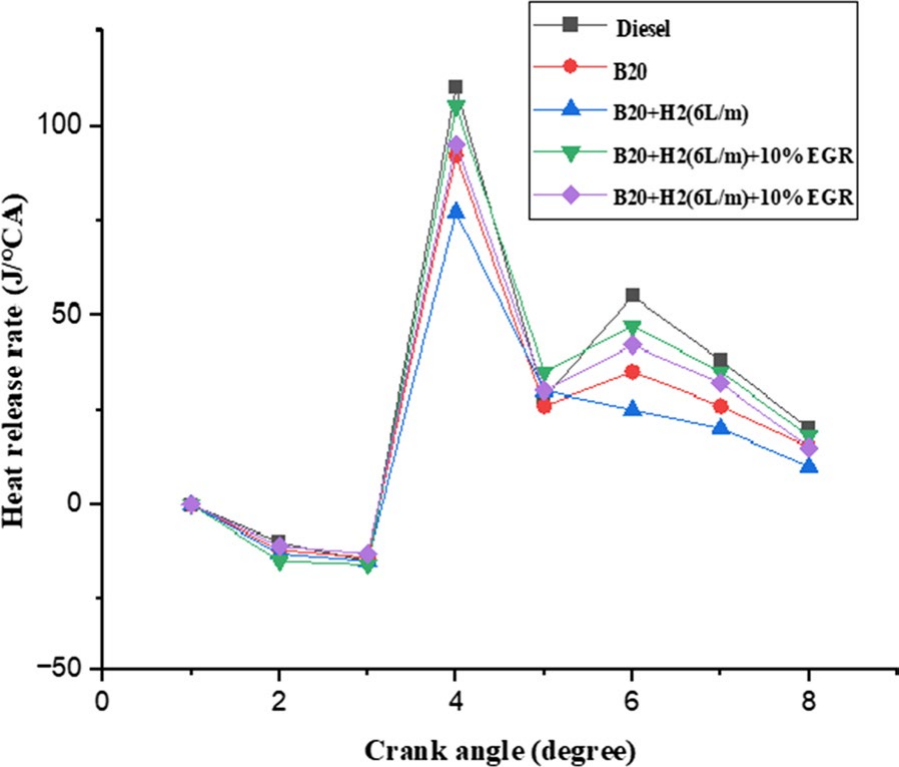
Effect of HRR with crank angle

Figure 11 depicts the curve for the heat release rate (HRR) with crank angle for different percentages of EGR addition with B20H2 (6L/m) dual fuel mode. It is clear to observe that 65.4 J/deg was the HRR with the highest value. As opposed to 47.64 J/deg CA for the dual fuel mode using B20H2 (6L/m). When using 20% EGR. It has been determined that the amount of heat released when the fuel was enriched with hydrogen was not increased by the addition of EGR. The HRR is held responsible for the high peak pressure that results from premixed combustion. Two primary parameters that affect the combustion processes in EGR operation are the calibre of the pilot biodiesel fuel spray and the mixing of the hydrogen and EGR in the cylinder charge. Due to the dilution effect during the premixed combustion phase, EGR decreased the HRR. This restricts the turbulent flame propagation from the pilot ignite zones to the cylinder charge. It also affects the amount of heat released during combustion [43].

## Conclusions

The experiment used a diesel engine of the Kirloskar, TV1 brand on B20 with the hydrogen enrichment with EGR technology. We used a constant engine speed of 1500 rpm and a variable load as our operating parameters. In comparison to diesel fuel at full load, the BTE rises by 22.8% for hydrogen enrichment without EGR, but falls somewhat with EGR operation. When compared to diesel fuel, only hydrogen enhancement without EGR method lowers the engine's BSEC. When compared to diesel fuel, hydrogen enrichment causes increased EGT, however EGR substantially lowers EGT. While the introduction of hydrogen causes a reduction in CO emissions, the use of EGR has little effect on those emissions. When hydrogen is introduced, UHC emissions at full load are reduced by 65.9% when compared to diesel fuel, but they are increased by 27.3% following EGR. With the EGR approach, NO_X_ emissions were significantly reduced by 25%, which can be attributed to the lower peak combustion temperature. In light of performance, emission, and combustion characteristics, the current study has demonstrated that induction of B20 with hydrogen on a single cylinder direct ignition CI engine with EGR technology is a workable engine operating approach.

## Data Availability

Data sharing is not applicable to this article as no new data were created or analysed in this research work.

## Abbreviations

CI: Compression ignition
NOx: Nitrous oxide
UHC: Unburned hydrocarbon
ppm: Parts per million
CO: Carbon monoxide
EGR: Exhaust gas recirculation
NOX: Oxides of nitrogen
TPI: Time port injection system
